# Supplementary material for: Epigenetic Regulation of MAP3K8 in EBV-Associated Gastric Carcinoma
Source: Int J Mol Sci. 2023 Jan 19;24(3):1964. doi: 10.3390/ijms24031964 (PMC9916342; doi:10.3390/ijms24031964)
Supplement: Supplementary file 1 [file ijms-24-01964-s001.zip › Migration assay_MAP3K8 siRNA treated YCCEL1 cells.pdf]

Figures 3K and 3L_YCCEL1 + MAP3K8 siRNA treatment

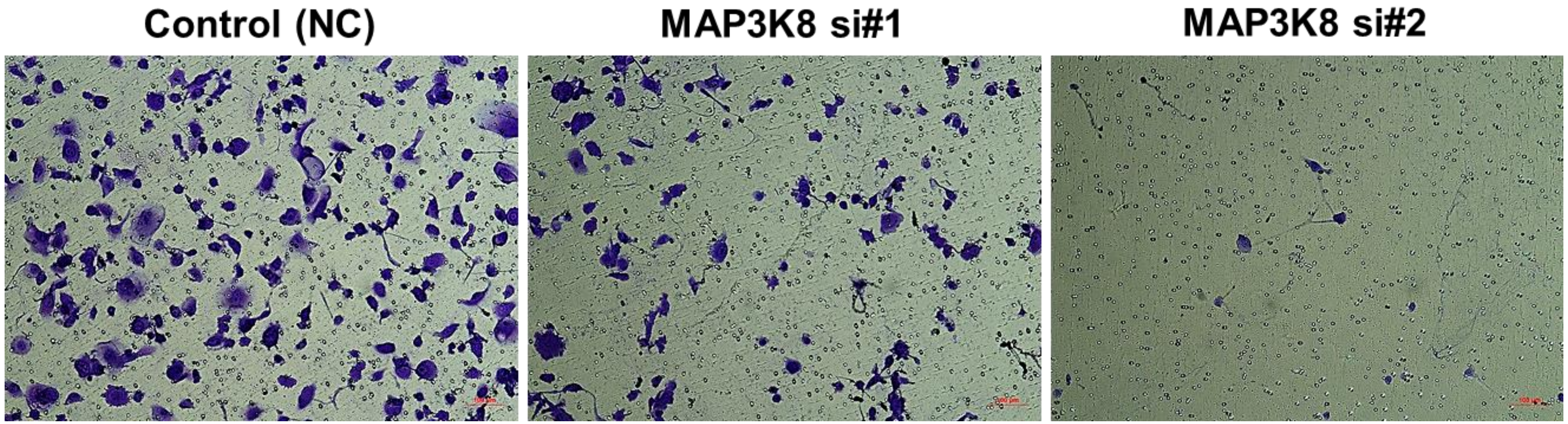

Pictures used: C1, si 1 2, and si 2 2

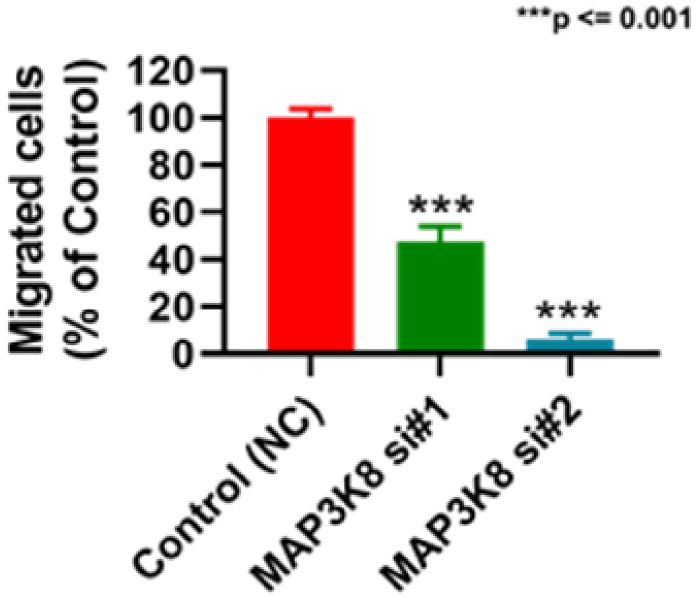

Figures 3K and 3L_YCCEL1 + MAP3K8 siRNA treatment_All 3 images

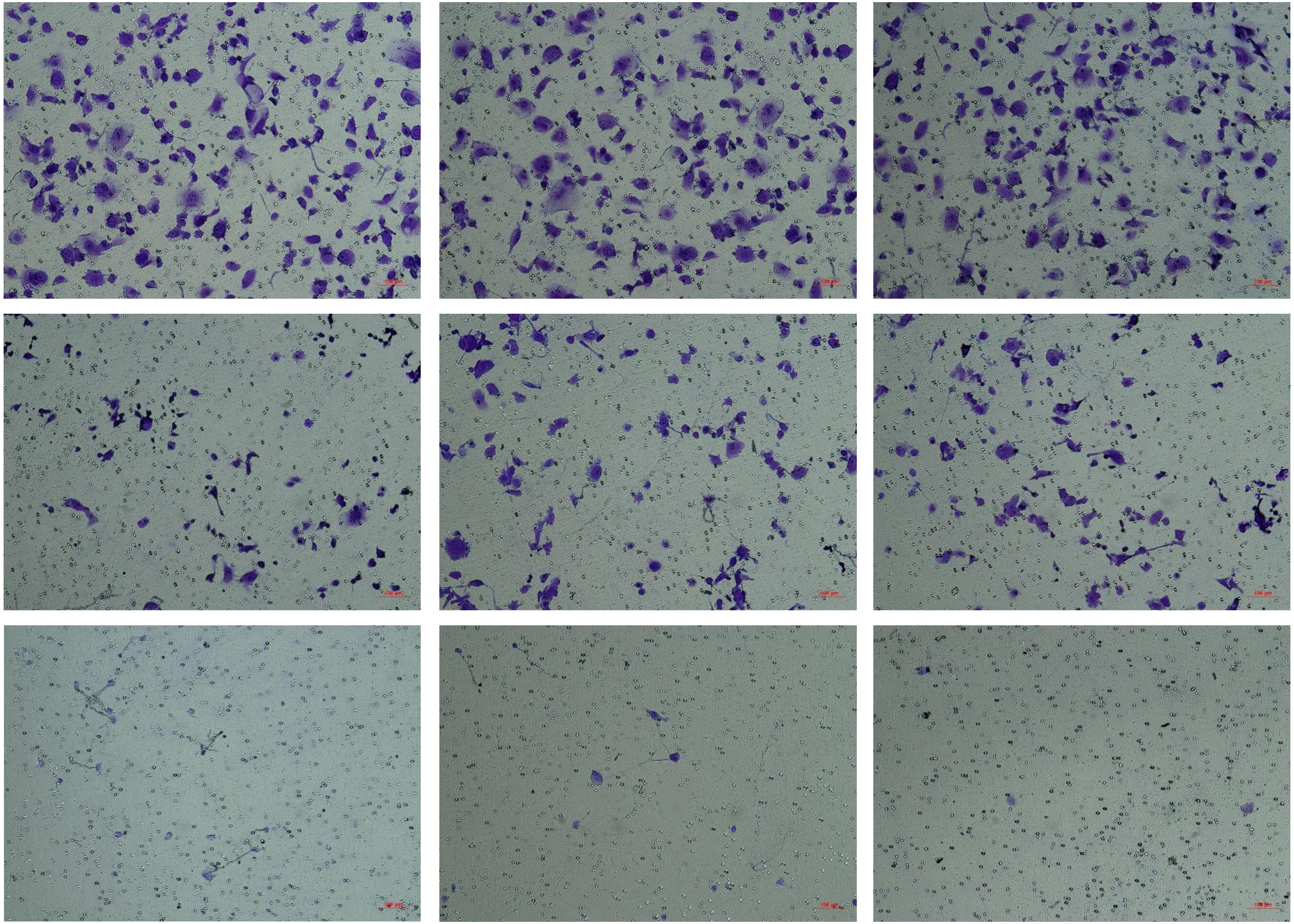

Control

MAP3K8 si#1

MAP3K8 si#2
