# Supplementary material for: Epigenetic Regulation of MAP3K8 in EBV-Associated Gastric Carcinoma
Source: Int J Mol Sci. 2023 Jan 19;24(3):1964. doi: 10.3390/ijms24031964 (PMC9916342; doi:10.3390/ijms24031964)
Supplement: Supplementary file 1 [file ijms-24-01964-s001.zip › Supplementary Figure S1.pdf]

Supplementary Figure S1:

Expression of *TGFBR1*, *SNAI1*, and *CTBP2* in stomach adenocarcinoma and its association with progression free survival (PFS) of GC patients: TCGA database was analysed using UALCAN portal to determine the expression of *TGFBR1*, *SNAI1*, and *CTBP2* in GC and normal patients. It was observed that the expressions of the above mentioned genes were significantly higher in STAD as compared to the normal samples (Figure S1A). Further, the association of these gene expressions were studied with respect to progression free survival of GC patients using the Kaplan Meier online portal and survival plots were constructed. The results indicated that expression of all three genes were inversely related to the disease/progression free survival of GC patients (Figure S1B).

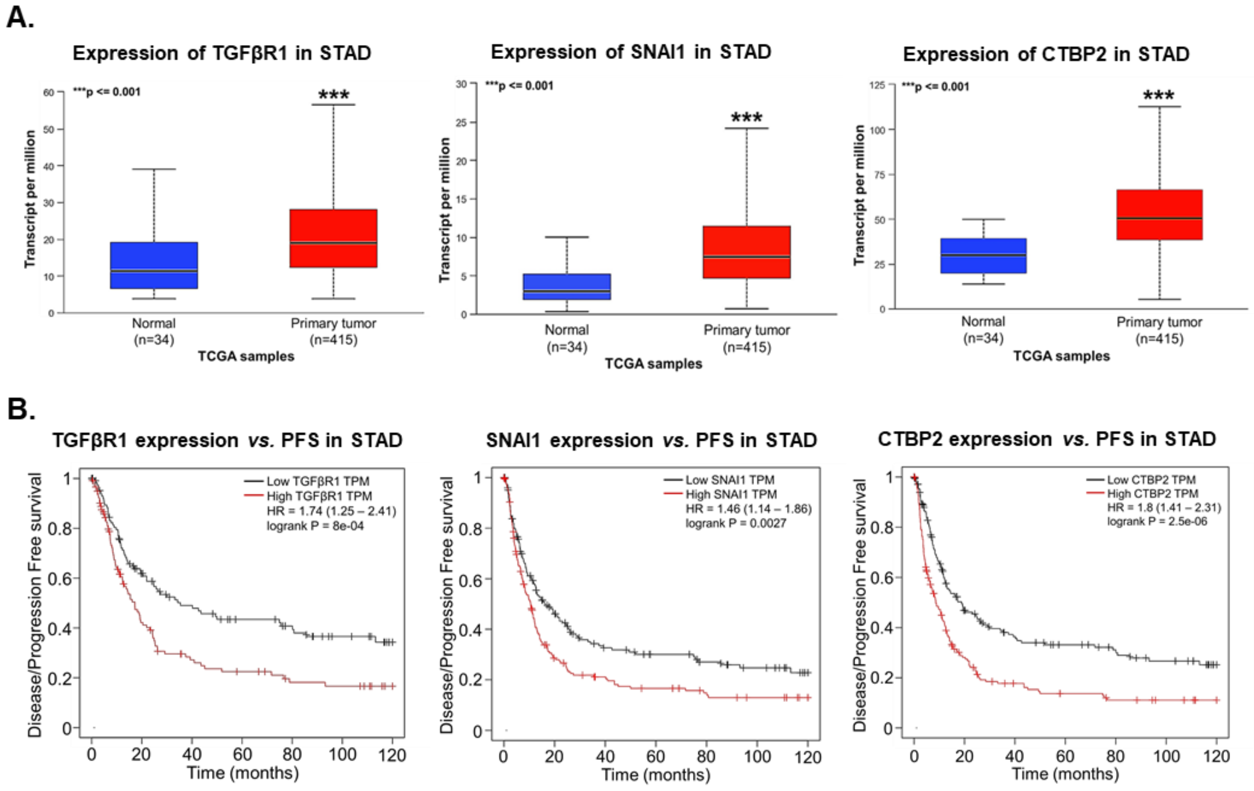

Supplementary Figure S1. Association between gene expression and progression free survival in STAD. (A) mRNA expression levels of *TGFβR1, SNAI1*, and *CTBP2* in GC tissues as compared to normal tissues. (B) Kaplan Meier plots *TGFβR1, SNAI1*, and *CTBP2 vs.* progression/disease free survival of GC patients. [***p <= 0.001].
