# Supplementary material for: Epigenetic Regulation of MAP3K8 in EBV-Associated Gastric Carcinoma
Source: Int J Mol Sci. 2023 Jan 19;24(3):1964. doi: 10.3390/ijms24031964 (PMC9916342; doi:10.3390/ijms24031964)
Supplement: Supplementary file 1 [file ijms-24-01964-s001.zip › Supplementary Tables.pdf]

Supplementary Table S1:

Sequence of primers used for making CRISPRi constructs.

Primer	5' → 3'
CRISPRi MAP3K8 SE Region 1 - F	CACCGTTAGTAACAGAAACCCCGCA
CRISPRi MAP3K8 SE Region 1 - R	AAACTGCGGGGTTTCTGTTACTAAC
CRISPRi MAP3K8 SE Region 2 - F	CACCGTGTCACGACAGTCCTGAGGT
CRISPRi MAP3K8 SE Region 2 - R	AAACACCTCAGGACTGTCGTGACAC
CRISPRi MAP3K8 SE Region 3 - F	CACCGGCTGGTGAGCTACCTCCCAA
CRISPRi MAP3K8 SE Region 3 - R	AAACTTGGGAGGTAGCTCACCAGCC
CRISPRi MAP3K8 SE Region 4 - F	CACCGTCAAACAGGTTAGGCCACCT
CRISPRi MAP3K8 SE Region 4 - R	AAACAGGTGGCCTAACCTGTTTGAC

Supplementary Table S2:

Sequence of primers used in qRT-PCR experiments.

Primer	5' → 3'
MAP3K8 Forward primer	ATGGAGTACATGAGCACTGGA
MAP3K8 Reverse primer	GCTGGCTCTTCACTTGCATAAAG
EpCAM Forward primer	AATCGTCAATGCCAGTGTACTT
EpCAM Reverse primer	CTCCTCCTGTTCGACAGTCAGC
SOX9 Forward primer	AGCCGACTCCCACATTTCCTCC
SOX9 Reverse primer	GAGATTGCCCAGAGTGCTCGCCC
TBX3 Forward primer	GCAGCTTTCAACTGCTTCG
TBX3 Reverse primer	TGAGGTTCGATGTCCCTACA

CXCL3 Forward primer	CGCCCAAACCGAAGTCAT
CXCL3 Reverse primer	GTGCTCCCCTTGTTCAGTATCT
ZFP36L1 Forward primer	ATGACCACCACCCTCGTGT
ZFP36L1 Reverse primer	TTTCTGTCCAGCAGGCAACC
DPYSL2 Forward primer	CCTTCCTCGTGTACATGGCTT
DPYSL2 Reverse primer	ATTTTCTGCGTGGACTTGGGCTA
SPINT2 Forward primer	AAGAATACTGCACCGCCAAC
SPINT2 Reverse primer	TTCTTCACCAGCTGCTCCTT
FZD5 Forward primer	ACACGCCCATCTGTCTGCC
FZD5 Reverse primer	CCTCGCTGCGGTTGTAATCC
SDC1 Forward primer	AAGATATCACCTTGTCACAGCA
SDC1 Reverse primer	GTTCTGGAGACGTGGGAATAG
SPRY1 Forward primer	CCCTGCCCTGGATAAGGAAC
SPRY1 Reverse primer	GGCCGAAATGCCTAATGCAA
TNFRSF11A Forward primer	TTGTGGCACTGGATCAATGAGG
TNFRSF11A Reverse primer	CCGTGAAGCACTGGCTTAAACTG
ITGB8 Forward primer	TGTGTGCTGGGCATGGAGAGTGT
ITGB8 Reverse primer	CAGTGCTGGGCTGCTGCTGAA
CLDN2 Forward primer	AACTACTACGATGCCTACC
CLDN2 Reverse primer	GAACTCACTCTTGACTTTGG
AHR Forward primer	CTCCCTCCACAGTTGGCTTTGTTTG
AHR Reverse primer	GATTCTGCGCAGTGAAGCATGTCAG
FAT1 Forward primer	GTGTTTGTTCTCTGCCGTAAG
FAT1 Reverse primer	TAGGCTTCTGGATGGAGTCG

SPRY4 Forward primer	CTGACCAACGGCTCTTAGAC
SPRY4 Reverse primer	GATGCACACTCCTTGCATTTAC
TBC1D16 Forward primer	AGATGCAGCTCAAAGACCAGG
TBC1D16 Reverse primer	GAAAATGGCCTTCCGCAGC
FERMT1 Forward primer	AAGATGGTGAGGTTGCGAGTC
FERMT1 Reverse primer	ATGGTGGATGATGCTGGTGTT
MYB Forward primer	CAGGAAGGTTATCTGCAGGAGTCTTCAAAA
MYB Reverse primer	CTATAGGCGGAGCCTGAGCAAAA
LBR Forward primer	TTCATGCTGGCTTTTGGAG
LBR Reverse primer	CTTTGGATCACTGGGATTTTTC
ZNF703 Forward primer	AACGGCCCACATGAGTCAAT
ZNF703 Reverse primer	GGCGGGGATCATGTCGTTAT
CLDN4 Forward primer	CGGCCCACAACATCATCCAA
CLDN4 Reverse primer	GGCGGAGTAAGGCTTGTCT
EPHB4 Forward primer	TCAATGTCACCACTGACCGAGAGGTA
EPHB4 Reverse primer	GGTATTTGACCTCGTAGTCCAGCACA
SLC28A3 Forward primer	CCCTTCCAGAAAGCGTGACATCTC
SLC28A3 Reverse primer	TGTTGATGAGTATGCCTGCGATGG
PTPN12 Forward primer	AGTTGCCTTGTTGAAGGGGAT
PTPN12 Reverse primer	AGAAGGTGTCAAGATGGGTGG
Notch1 Forward primer	CGTGCTATGTTGTGGACCATGGC
Notch1 Reverse primer	CACACTCGTGGGTGGTGTCCCCCG
Notch2 Forward primer	TCAACTGCCAAGCGGATGT
Notch2 Reverse primer	CTTGGCTGCTTCATAGCTCC

CTBP2 Forward primer	ACTGTGGCCTTCTGTGACGC
CTBP2 Reverse primer	CTGGTGAGGGTGATGGTGTG
ACTA2 Forward primer	TCTGGCACCACTCTTTCTAT
ACTA2 Reverse primer	CATGAGGTAGTCGGTGAGAT
TGFBR1 Forward primer	TGTCAAAGTCAGTCCGTTGGGTCT
TGFBR1 Reverse primer	ACTTTGAAAGCCACACAAGCCCTG
SNAI1 Forward primer	TCGGAAGCCTAACTACAGCGA
SNAI1 Reverse primer	AGATGAGCATTGGCAGCGAG
SNAI2 Forward primer	CGAACTGGACACACATACAGTG
SNAI2 Reverse primer	CTGAGGATCTCTGGTTGTGGT
GAPDH Forward primer	GTCTCCTCTGACTTCAACAGCG
GAPDH Reverse primer	ACCACCCTGTTGCTGTAGCCAA
