# Supplementary material for: Epigenetic Regulation of MAP3K8 in EBV-Associated Gastric Carcinoma
Source: Int J Mol Sci. 2023 Jan 19;24(3):1964. doi: 10.3390/ijms24031964 (PMC9916342; doi:10.3390/ijms24031964)

Figure 2F_WB_MAP3K8_OTX015 treated SNU719 cells

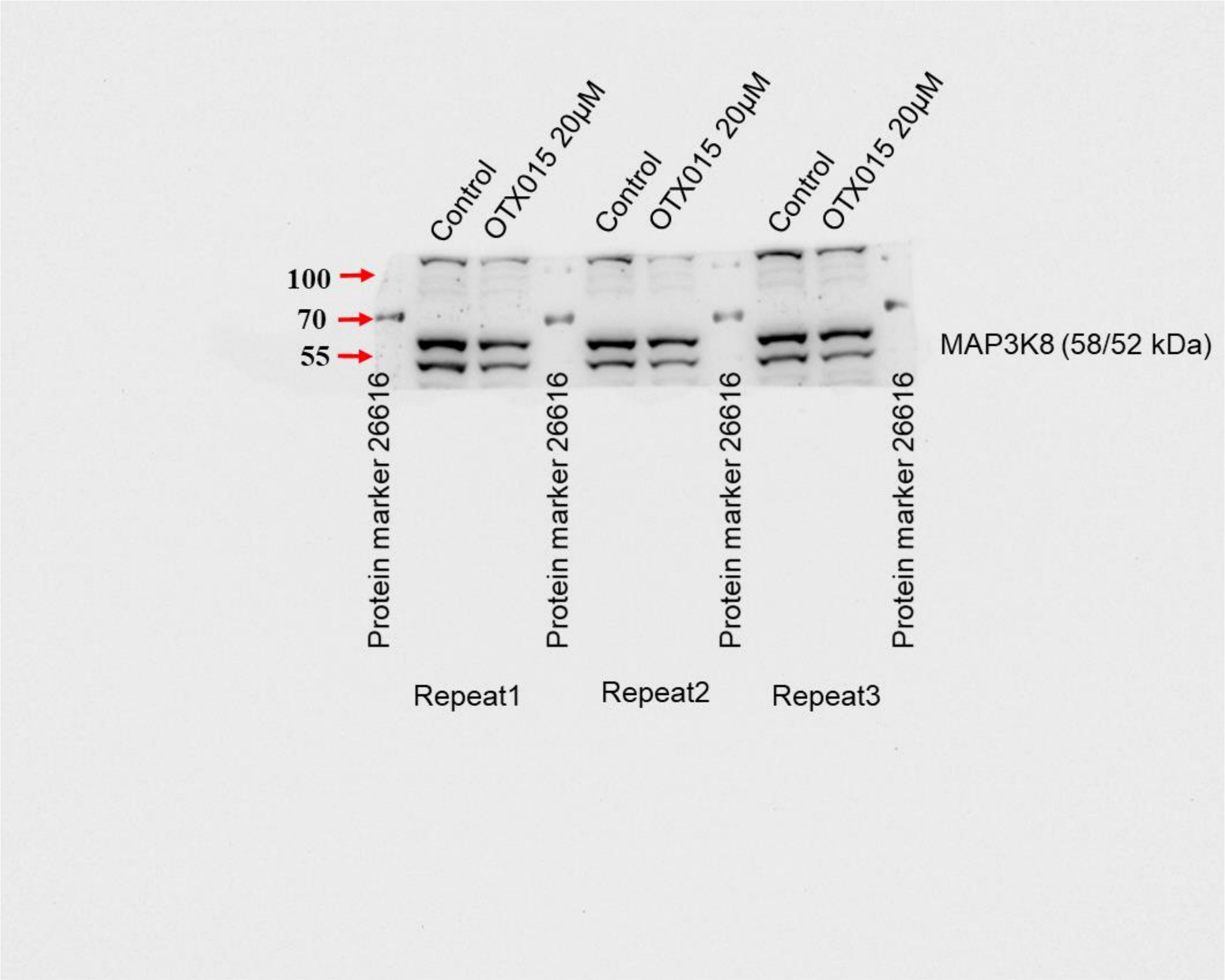

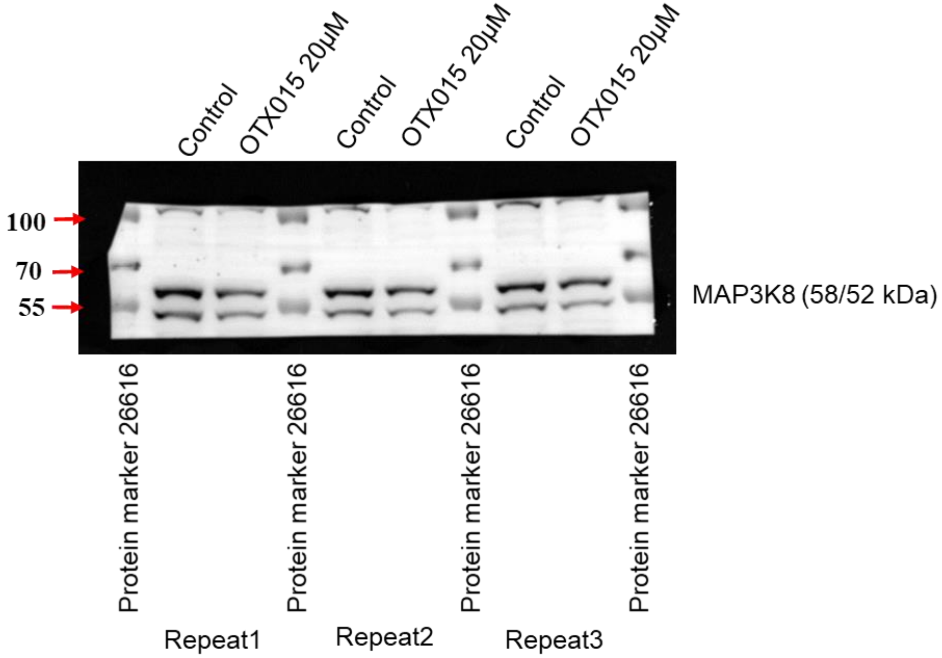

Figure 2F_WB_Actin_OTX015 treated SNU719 cells

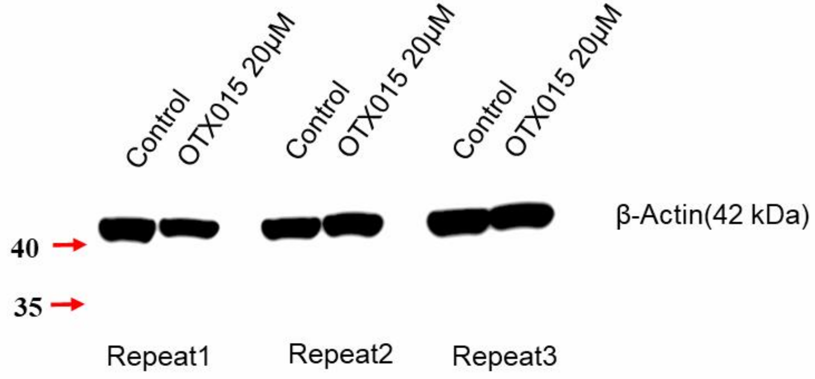

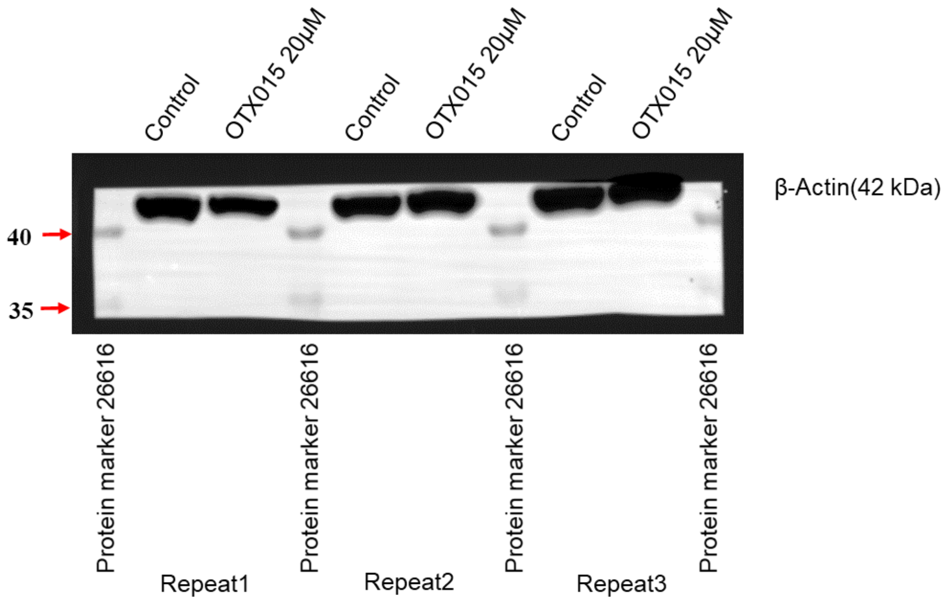

Figure 2I_WB_MAP3K8_OTX015 treated YCCEL1 cells

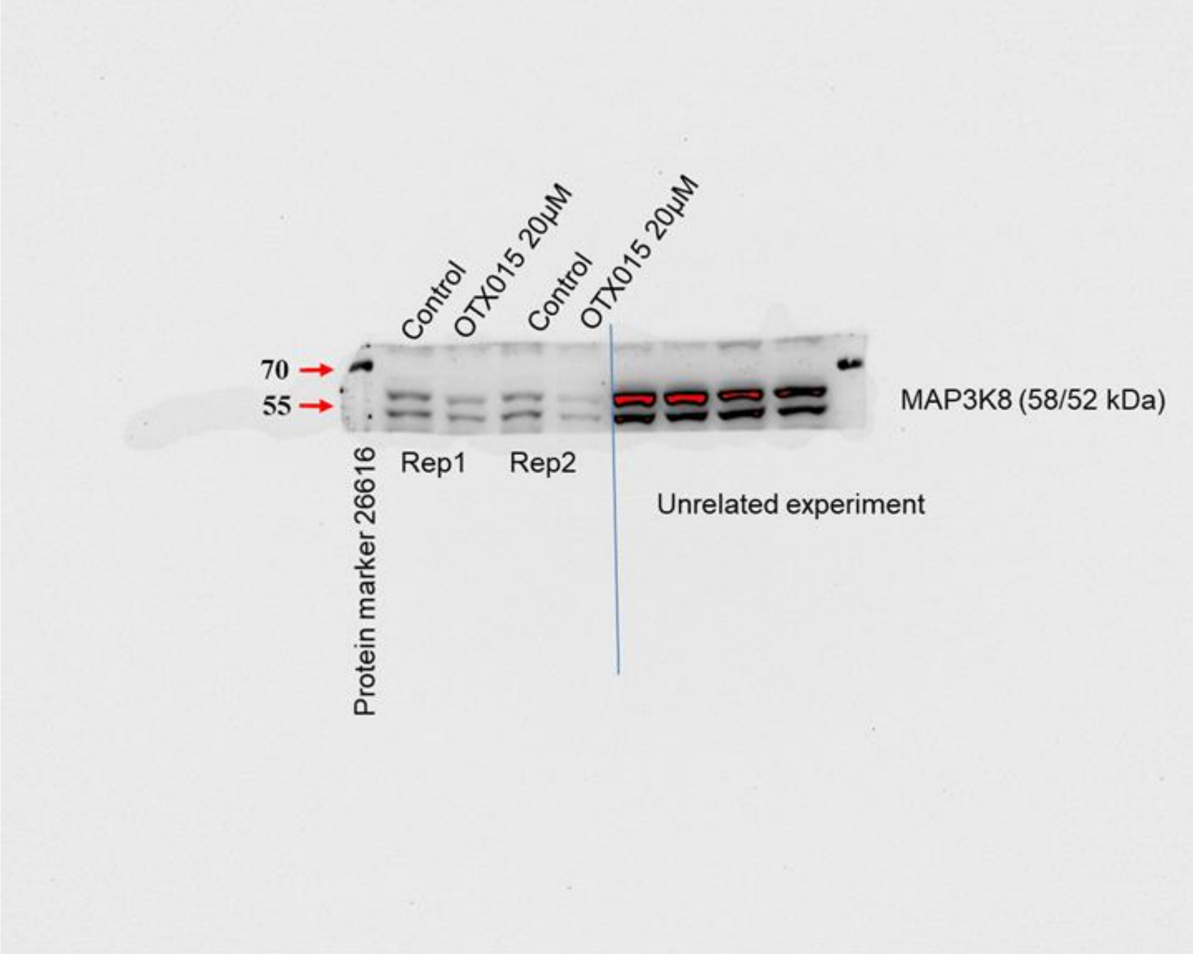

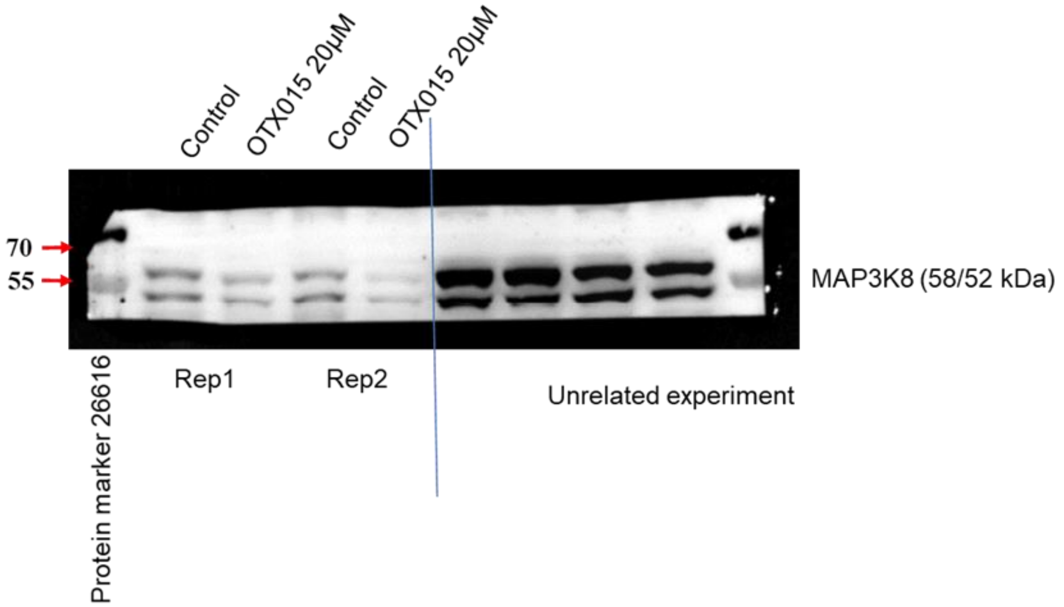

Figure 2I_WB_Actin_OTX015 treated YCCEL1 cells

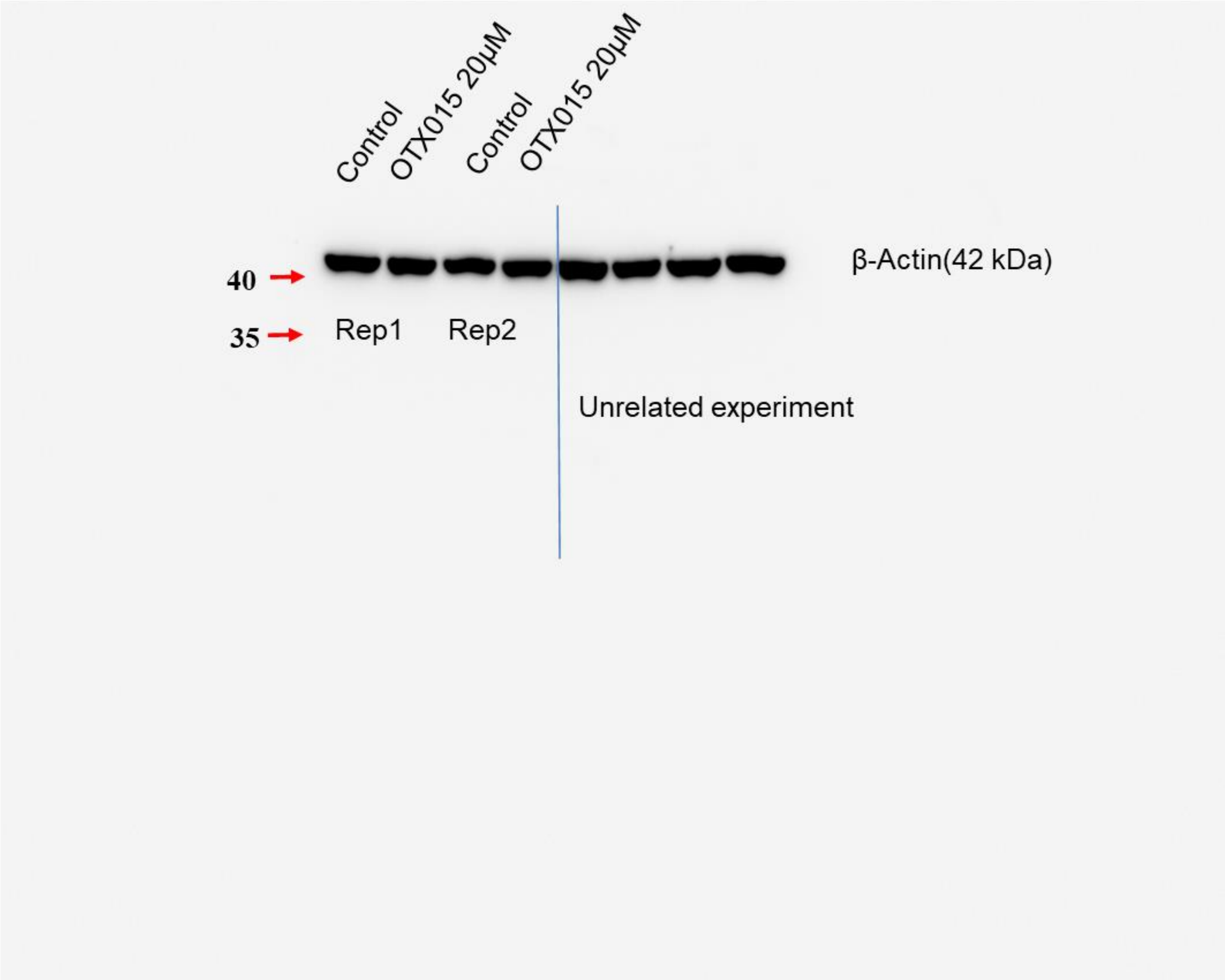

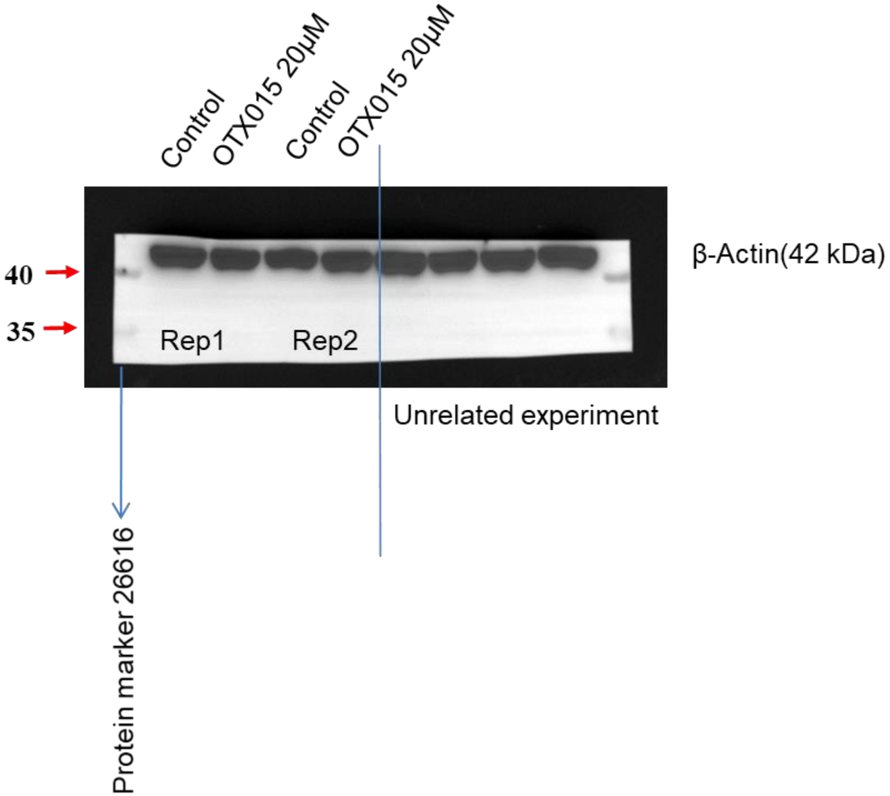

Figures 3A and 3C_WB_MAP3K8_MAP3K8 siRNA treated EBVaGC cells

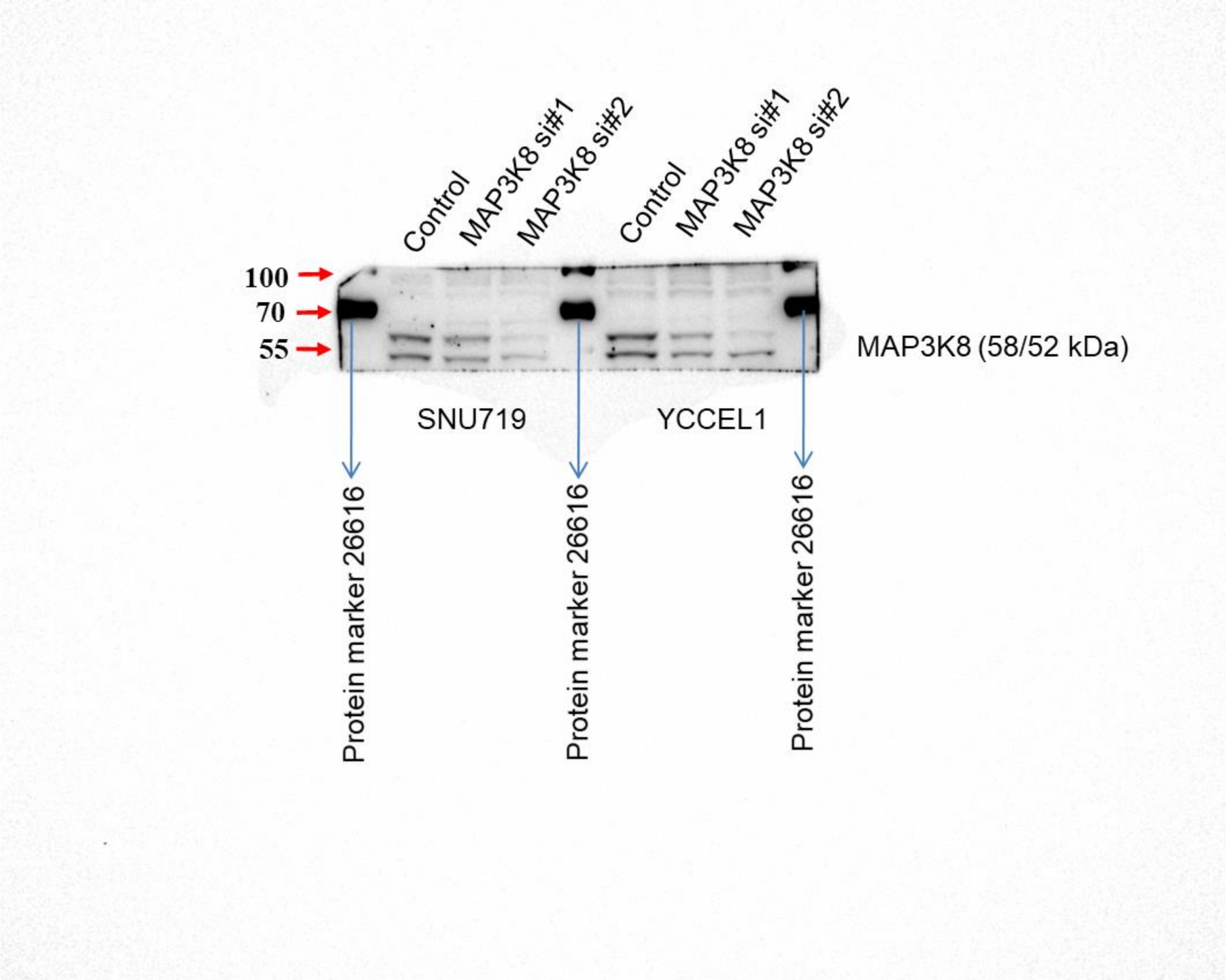

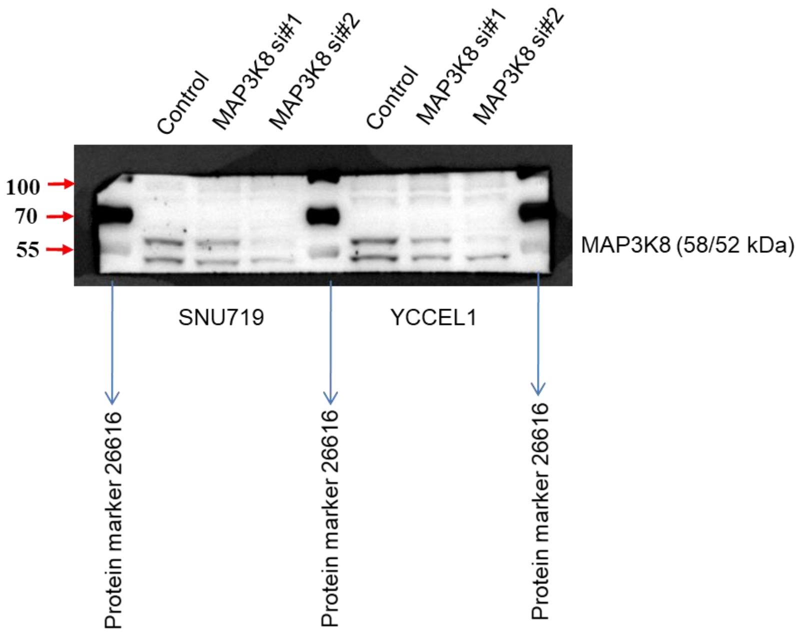

Figures 3A and 3C_WB_Actin_MAP3K8 siRNA treated EBVaGC cells

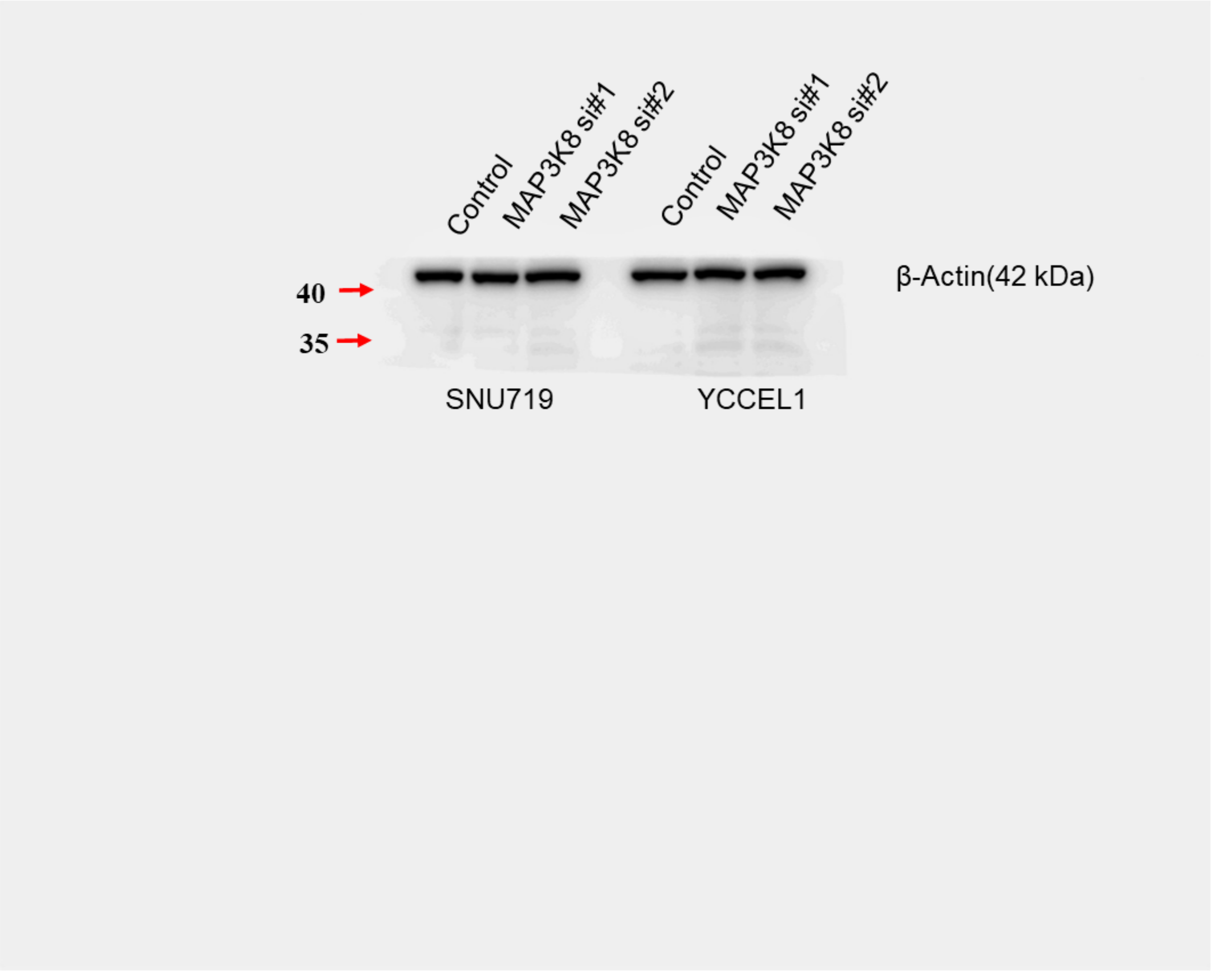

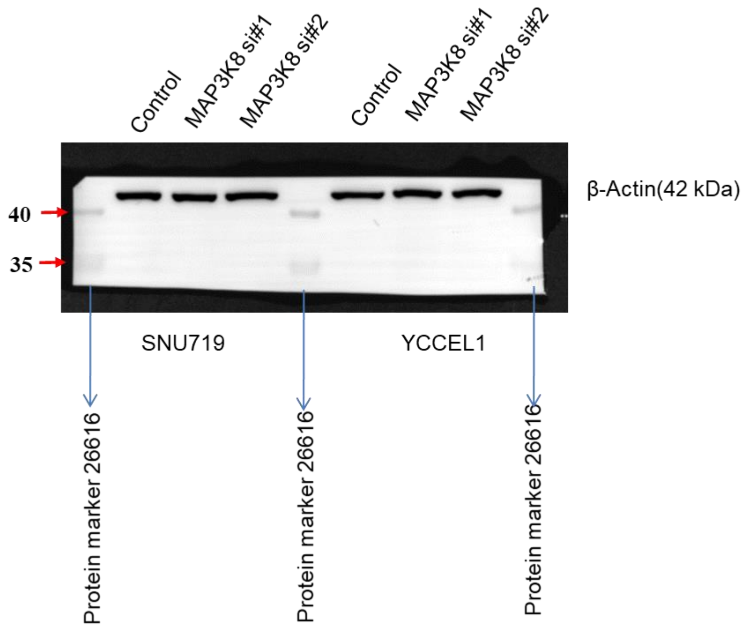

Supplement: Supplementary file 1 [file ijms-24-01964-s001.zip › WB_Epigenetic regulation of MAP3K8 in EBV-associated Gastric Carcinoma.pdf]
